# ID 250 - Custo-Efetividade e Análise de Impacto Orçamentário dos Transplantes Intestinal e Multivisceral em Pacientes com Falência Intestinal

**DOI:** 10.5327/2237-9622.2025.v34s1.250

**Published:** 2025-11-25

**Authors:** Roberta Crevelário de Melo, Bruna Carolina de Araújo, Letícia Aparecida Lopes Bezerra da Silva, Cinthia Lanchotte Ferreira, Thaiana Helena Roma Santiago, Antonio Pescuma, Juliana Abud, Alessandra Crescenzi, Alexandre Chagas de Santana, Cláudia Lima Vieira, Inara Pereira da Cunha, Márcia Saldanha Kubrusly, Alex Jones Flores Cassenote, Wallace Breno Barbosa, Luciana Costa Xavier, Luciana Bertocco de Paiva Haddad

**Keywords:** transplante de intestino, transplante multivisceral, falência intestinal, custo-efetividade, análise de impacto orçamentário

## Abstract

**Introdução:**

A falência intestinal (FI) é uma condição rara, caracterizada pela incapacidade do órgão em manter a digestão e a absorção de nutrientes necessários para a manutenção nutricional do indivíduo. Estima-se que sua prevalência seja em torno de 20 a 80 casos por milhão de adultos e de 14,1 a 56 casos por milhão de crianças. Entre a população com diagnóstico de FI, 10% a 15% serão candidatas ao transplante de intestino delgado (TID) e ao transplante multivisceral (TMV). O TID e o TMV são procedimentos indicados para pacientes com FI irreversível que correm risco de morte por causa de complicações relacionadas à nutrição parenteral total (NPT). Dessa forma, este estudo tem como objetivo avaliar a custo-efetividade e a análise do impacto orçamentário (AIO) do TID ou do TMV comparados à nutrição parenteral total em paciente com FI.

**Método:**

Elaborou-se uma avaliação econômica de custo-efetividade e uma análise do impacto orçamentário dos dois procedimentos na população estudada na perspectiva do Sistema Único de Saúde (SUS). Foi realizada uma avaliação para estimar a relação de custo-efetividade incremental (RCEI) entre TID em relação à NPT na FI. O desfecho avaliado para medir a efetividade das tecnologias foi a sobrevida do paciente. Foram considerados apenas os custos médicos diretos referentes aos procedimentos, cuidados pré-transplante e pós-transplante, cujos valores foram provenientes do sistema de convênio que a saúde pública brasileira tem, atualmente, com hospitais de referência para realização do procedimento. Na análise de impacto orçamentário, foi considerado um horizonte temporal de cinco anos; e, para a composição do cenário atual, sem a incorporação das novas tecnologias, foi considerada a NPT como opção. Para o cálculo da população elegível para o primeiro ano de análise, foi considerada a prevalência da falência intestinal com indicação de transplantes em casuísticas internacionais, além da disponibilidade de órgãos para transplantes no Brasil. Foram considerados os mesmos custos utilizados na análise de custo-efetividade.

**Resultados:**

Como resultado, a RCEI foi de R$ 1.201.028,47 por ano acrescido à sobrevida do paciente. Os resultados das análises de sensibilidade probabilística mostraram ampla variação de RCEI, entre R$ 321.934,23 e R$ 7.405.729,85. Em relação a AIO, observou-se que a incorporação do TID para pacientes com FI no SUS resultaria num incremento de custo de R$ 13.254.038,51 no primeiro ano e de R$ 22.539.058,50 no quinto ano.

**Conclusão:**

O TID e o TMV para FI apresentam uma razão de custo incremental bastante elevada em relação aos comparadores, porém devem ser considerados num cenário de raridade da indicação e do acesso ao procedimento, devendo ser consideradas as imprecisões na avaliação do custo do procedimento. A análise do impacto orçamentário demonstra que ambos os cenários apresentam custos maiores que o cenário atual nos primeiros cinco anos após o transplante. Contudo, dependendo dos valores do procedimento, os custos podem ser semelhantes ou menores em comparação ao cenário atual.

